# Preparation of multi-scale FOX-7 particles and investigation of sensitivity and thermal stability†

**DOI:** 10.1039/c9ra03394g

**Published:** 2019-07-04

**Authors:** Ruixuan Xu, Chongwei An, Hao Huang, Jingyu Wang, Baoyun Ye, Bin Liu

**Affiliations:** School of Environment and Safety Engineering, North University of China Taiyuan 030051 Shanxi China anchongwei@yeah.net; Shanxi Engineering Technology Research Center for Ultrafine Powder, North University of China Taiyuan 030051 Shanxi China; China North Industries Group Corporation Limited Beijing 100821 China

## Abstract

Multi-scale ultrafine 1,1-diamino-2,2-dinitroethene (FOX-7) samples with different particle size were fabricated and specifically, nano-FOX-7 was successfully prepared by a green mechanophysical milling method. All samples were characterized by field emission scanning electron microscopy (FE-SEM) and X-ray diffraction (XRD). Impact and friction sensitivities of the samples were tested and thermal analysis was performed by differential scanning calorimetry (DSC) and thermogravimetry (TG). Ultrafine particles with a mean size of 40 nm, 0.9 μm and 3.4 μm respectively showed less sensitivity than raw FOX-7, whose particles size was about 20 μm. The critical drop height *H*_50_ of ultrafine FOX-7 increased from 129 cm to 172 cm, 142 cm and 136 cm, respectively and the friction sensitivity reduced from 32% to 8%, 16% and 20%, respectively. Furthermore, the apparent activation energy of ultrafine particles increased compared with raw materials, which suggested the thermal stability of the ultrafine particles was improved.

## Abstract 2

Multi-scale ultrafine 1,1-diamino-2,2-dinitroethene (FOX-7) samples with different particle size were fabricated and specifically, nano-FOX-7 was successfully prepared by a green mechanophysical milling method.

## Graphical abstract

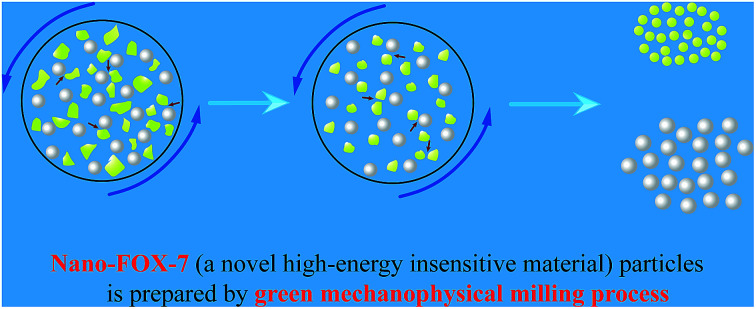

## 1 Introduction

1,1-Diamino-2,2-dinitroethene (C_2_H_4_N_4_O_4_, also known as FOX-7) is a novel high-energy insensitive material with a symmetrical molecular component and infinite 2D wave-shaped monolayer molecular packing, whose outstanding properties have attracted the attention of many scholars since it was first reported by Latypov in 1998.^1^ The special structure of FOX-7 determines its high thermal stability and low sensitivity.^2–5^ A large amount of literature has reported in detail the synthesis, molecular structure, thermal behavior and reactivity of FOX-7. Some scholars have studied the preparation methods of small-sized FOX-7. FOX-7 quasi-three-dimensional (3D) grids, which are constructed from one-dimensional nanostructures ∼100 nm in diameter, were synthesized by a spray freeze-drying technique and had a promising high-energy-density with superior sensitivity properties.^6^ Using a micellar nanoreactors, Mandal^7^ prepared spherical particles of FOX-7 with diameters generally in the submicrometer to nanometer range. Nano-FOX-7 was also prepared *via* an ultrasonic spray-assisted electrostatic adsorption (USEA) method, with an average particle size of approximately 78 nm and nano-FOX-7 particles showed faster energy release efficiency and release more energy.^8^

It was reported that when the particle size of the explosive is reduced to the nanometer size, a higher decomposition rate and a lower impact sensitivity are observed compared with the raw material.^9^ Furthermore, particle size was proved to have a significant role affecting the safety of the explosive.^10^ The safety of explosives is vitally important to their production, storage, transportation and use. Accordingly, the effect of FOX-7 particle size on its performance and safety is investigated in this paper.

The ultrafine method of explosive particles mainly includes physical methods^11–14^ and chemical methods.^15–17^ Compared with chemical methods, mechanophysical milling, a completely physical method, will not cause environmental pollution and more importantly, is low cost and mass production can be achieved. A series of experiments have proved that mechanophysical milling is an effective method for preparing ultrafine explosive particles. Liu *et al.*^11^ prepared nano-HMX with different particle size (120.36 μm, 1.18 μm and 0.16 μm) using a bi-directional rotation mill under different drying conditions and there was a significant sensitivity decrease for 0.16 μm HMX particles. Wang *et al.*^12^ used a mechanical milling approach to fabricate HMX nanoparticles with a significant proportion of nano-HMX (<100 nm) which was far less sensitive than raw HMX. In addition, mechanophysical milling method was widely adopted to produce other nanoscale explosive particles such as CL_20 (with an average size of 200 nm ^13^ and 73.8 nm,^14^ respectively), PETN^14^ (with a mean size of 267.7 nm), HNS^18^ (about 89.2 nm with a narrow size distribution), HMX/TATB explosive cocrystals^19^ (about 100–300 nm in size), CL_20/HMX explosive cocrystals^20^ (with an average size below 200 nm) and all test results revealed that ultrafine explosive particles were less sensitive than raw materials.

Herein, mechanophysical milling method was adopted to fabricate ultrafine FOX-7 particles. In order to prevent possible explosion, the preparation process of the ultrafine explosive particles is performed in a liquid medium, which is an aqueous suspension of explosive particles mixing with bead. Moreover, the mechanical and thermal safety of raw and ultrafine FOX-7 were investigated and compared in this paper.

## 2 Experimental section

### 2.1 Materials and equipment

Raw FOX-7 (1,1-diamino-2,2-dinitroethene, C_2_H_4_N_4_O_4_, the purity = 99.71%) was bought from Gansu Yinguang Chemical Industry Group Co., Ltd. (Baiyin city, P. R. China). Absolute ethyl alcohol (C_2_H_5_OH, AR grade) and dimethyl sulfoxide (C_2_H_6_OS, AR grade, abbreviated as DMSO) were purchased from Tianjin Guangfu Technology Development Co., Ltd (Tianjin city, P. R. China). Pure water (distilled water) was obtained from our own laboratory. Zirconia ball was obtained from Shandong Zibo Yubang Industrial Ceramics Co., Ltd (Zibo city, Shandong province, P. R. China). MITR-YXQM-1L ball mill was purchased from Changsha MITR Instrument and Equipment Co., Ltd (Changsha city, P. R. China).

### 2.2 Preparation

**Fig. 1:**
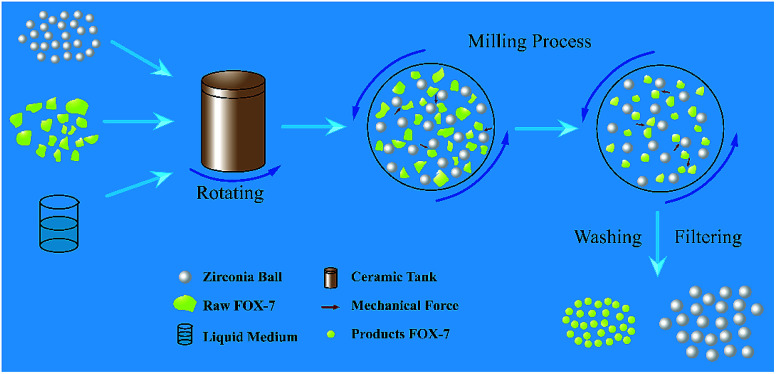
Schematic diagram of mechanophysical milling.

Mechanophysical milling was used to fabricate ultrafine FOX-7 particles under different experimental conditions and the schematic diagram of mechanophysical milling is shown in Fig. 1. The same quality raw FOX-7 and zirconia ball were added into two ceramic tanks respectively (the mass ratio of raw FOX-7 to zirconia ball was 1 : 20). There was a difference of the liquid medium in two tanks: (1) the liquid medium was pure water which was 10 times the mass of raw FOX-7; (2) the liquid medium was a mixture of pure water and absolute ethyl alcohol and the mass ratio of pure water and ethyl alcohol to raw FOX-7 is 5 : 1 respectively. The milling conditions: 350 rpm and rotated continuously for six hours. The solid explosive particles were pulverized into small particles under the action of mechanical forces and a yellow mixed suspension of zirconia ball, liquid medium and as-prepared explosive particles was obtained. After washing, filtration and freeze-drying, two kinds of FOX-7 particles were obtained from two tanks (marked as FOX-7-1 and FOX-7-2, respectively). (Please pay attention to the potential danger during milling process and the corresponding safety precautions are necessary.)

As a comparison, other FOX-7 particles were prepared by a recrystallization method. FOX-7 is readily dissolved in DMSO and essentially insoluble in water.^4^ Therefore, we used DMSO as solvent and pure water as non-solvent. 5 g of FOX-7 was dissolved in 32 ml of DMSO to prepare FOX-7/DMSO solution. This is a prefilming twin-fluid nozzle-assisted precipitation method.^15,16^ Pure water was pumped into a nozzle which is a venturi tube and FOX-7/DMSO solution was sucked into the nozzle under negative pressure caused by high speed flow of pure water. The explosive particles crystallize out during the mixing process of two fluids which were DMSO and pure water. The mixture of explosive particles and solution was collected in a beaker and FOX-7 products was obtained after filtration and freeze-drying (marked as FOX-7-3) (Table 1).

**Table 1:** Labels of FOX-7 products

Sample	Method	Solvent	Nonsolvent
FOX-7-1	Mechanophysical milling	—	Pure water
FOX-7-2	Mechanophysical milling	—	Pure water + absolute ethyl alcohol
FOX-7-3	Recrystallization	DMSO	Pure water

### 2.3 Characterization

#### 2.3.1 Field emission scanning electron microscopy (FE-SEM)

The microstructure and morphology of raw FOX-7 and products were characterized by field emission scanning electron microscopy (FE-SEM, JSM-7500F, JEOL, Japan) at an acceleration voltage of 5 kV after gold sputtering coating. The size distribution of raw FOX-7 and FOX-7-1 was characterized using laser diffraction particle size analyzer (90 PLUS, Brookhaven Instruments Corporation, New York, America).

#### 2.3.2 X-ray diffraction

X-ray diffraction (XRD, DX-2700, Dandong Haoyuan Corporation, Liaoning, China) analysis was used to test the crystalline data of raw FOX-7 and products with Cu-Kα radiation at 40 kV and 30 mA. And the samples were scanned from 5° to 50° in 2*θ*, with an increment of 0.05° and a scan speed of 0.5 s per step.

#### 2.3.3 Impact sensitivity test

The impact sensitivity of raw FOX-7 and products was tested by a Type 12 drop-hammer apparatus. Test conditions: ambient temperature: 20–25 °C; relative humidity: 60%; sample mass: (30 ± 1) mg; drop hammer mass: (2 ± 0.002) kg.

#### 2.3.4 Friction sensitivity test

The friction sensitivity of raw FOX-7 and products was measured by a WM-1 type friction sensitivity apparatus. Test conditions: ambient temperature: 20–25 °C; relative humidity: 60%; sample mass: (20 ± 1) mg; swing angle: 90°; gauge pressure: (3.92 ± 0.07) MPa.

#### 2.3.5 Differential scanning calorimetry and thermogravimetry

All samples were analyzed by a differential scanning calorimetry (DSC) under the following conditions: sample mass: 0.5 mg; heating rate: 5 °C min^−1^, 10 °C min^−1^, 15 °C min^−1^ and 20 °C min^−1^; nitrogen atmosphere. Thermal gravimetric analysis (TGA1, Mettler Toledo, Switzerland) was carried out in the nitrogen atmosphere with a heating rate of 20 °C min^−1^.

## 3 Results and discussions

### 3.1 Size and morphology

**Fig. 2:**
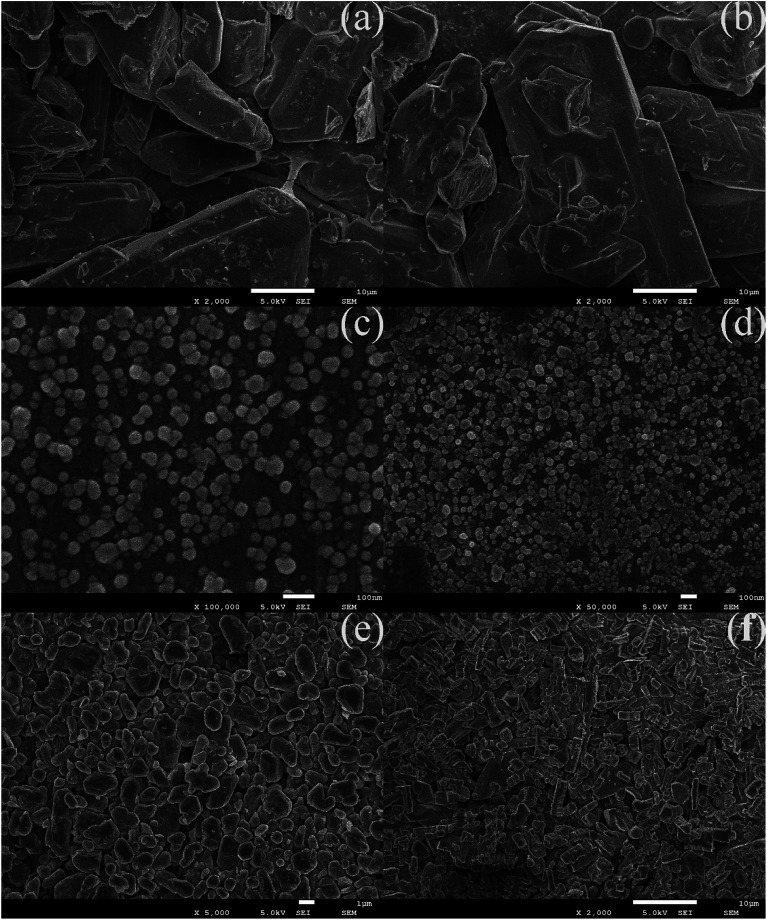
SEM images of explosive particles: (a) and (b) raw FOX-7, (c) and (d) FOX-7-1, (e) FOX-7-2, (f) FOX-7-3.

**Fig. 3:**
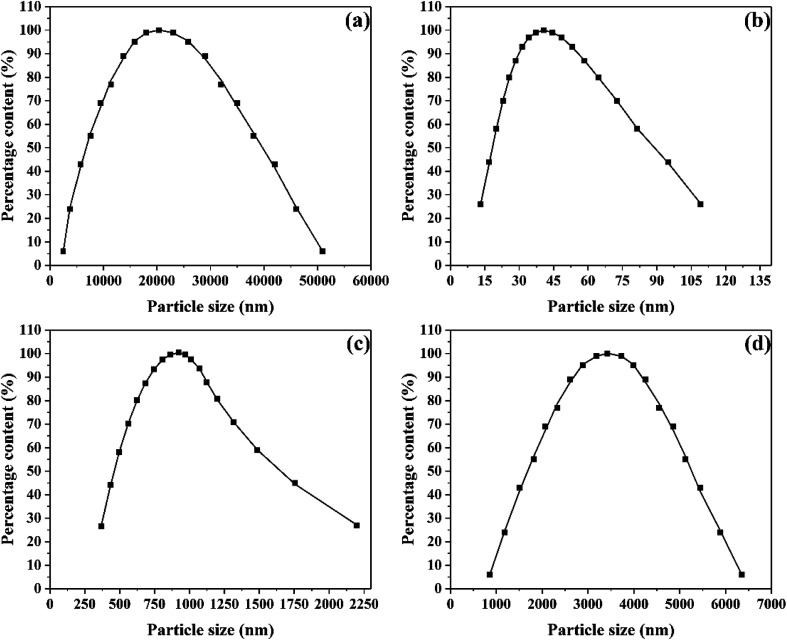
Particle size distribution: (a) raw FOX-7, (b) FOX-7-1, (c) FOX-7-2 and (d) FOX-7-3.

The microstructure and morphology of raw and ultrafine FOX-7 were observed by FE-SEM characterization (as shown in Fig. 2) and the particle size distribution curves of raw FOX-7 and products are shown in Fig. 3. The horizontal axis in Fig. 3 indicates the particle size (nm) and the vertical axis indicates the relative percentage share of particle size (%). There are obvious differences in morphology and particles size between raw FOX-7 and products. We can see that raw FOX-7 had an irregular prismatic and massive shape with conspicuous edges and corners (Fig. 2a and b), while FOX-7-1 had a regular ellipsoidal shape (Fig. 2c and d). The particle size of raw FOX-7 was between 2–50 μm (as seen in Fig. 3a) and FOX-7-1, as-prepared ultrafine particles, had a relatively narrow size distribution and the median size was about 40 nm, ranging from 15 nm to 110 nm (Fig. 3b). Furthermore, FOX-7-2, produced by mechanophysical milling in water and absolute ethyl alcohol, had a massive and ellipsoidal shape and had a mean particle size of 0.9 μm, ranging from 0.3 μm to 3 μm (as shown in Fig. 2e and 3c). As a comparison, FOX-7-3, prepared by recrystallization, had several different shapes including cubic and rod-shaped mostly with obvious edges and corners, whose mean size was about 3.4 μm, ranging from 0.9 μm to 6.5 μm (as shown in Fig. 2f and 3d). Therefore, multi-scale FOX-7 particles with different morphologies were successfully fabricated by mechanophysical milling and recrystallization process.

It has been observed that raw FOX-7 and products were both yellow while raw FOX-7 was darker than products. Raw FOX-7 may contain trace impurities which make it darker. These impurities were dissolved in the solution during recrystallization process and were filtered out, which made the recrystallized product lighter in color. Besides, ultrafine particles prepared by mechanophysical milling is slightly lighter in color than that prepared by recrystallization method. Compared with recrystallization process, the extrusion, friction, and collision between the explosive particles and the rapid rotation during the grinding cause the particles to fade in color.

During mechanophysical milling process, the edges and corners of the explosive particles are gradually smoothed by the strike generated between explosive particles and zirconia balls. Under the action of mechanical force, the explosive particles are continuously subjected to extrusion, shear, collision and friction, so that the explosive particles are pulverized into ultrafine particles undergoing three stages of crack formation, expansion and fracture. So ellipsoidal and nanoscale particles were obtained through continuous grinding. Ethyl alcohol is the only difference between the fabrication process of FOX-7-1 and FOX-7-2, whose particle size obviously differed. The process of rapid grinding assisted dissolution of FOX-7 in absolute ethyl alcohol and the bigger particle size resulted from growth of crystals in absolute ethyl alcohol.

During the recrystallization process, when FOX-7/DMSO solution and the pure water are rapidly mixed, the solution suddenly reaches to a supersaturation state and fast nucleation occurs, then the explosive particles are crystallized out of the solution. Due to the high-speed fluid and rapid mixing process, crystal nucleation and growth of explosive particles are not easy to control, resulting in the observation of particles with different sizes and morphologies.

### 3.2 XRD analysis

**Fig. 4:**
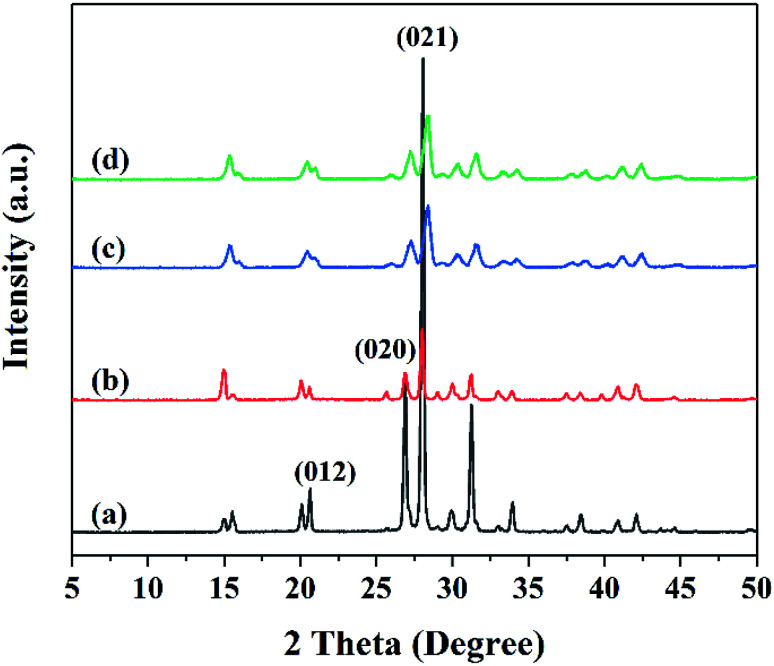
X-ray diffraction patterns: (a) raw FOX-7, (b) FOX-7-1, (c) FOX-7-2, (d) FOX-7-3.

The XRD patterns of raw FOX-7 and products are shown in Fig. 4. Raw FOX-7 displays three typical peaks at 2*θ* = 20.654°, 26.900°, 28.066° which are assigned to the (012), (020), (021) reflection lines. The XRD patterns of products in Fig. 4(b–d) are consistent with that of raw FOX-7 in Fig. 4(a). As we can see from the patterns, FOX-7 products had weaker diffraction peaks than raw FOX-7 and the diffraction peaks were broadened, which is due to the reduction of particle size.^21^ The patterns of products in Fig. 4(b–d) have similar diffraction angles compared with the raw FOX-7 shown in Fig. 4(a), indicating that the ultrafine particles were still FOX-7 and the crystal form didn't change during the process of mechanophysical milling and recrystallization process.^22^

### 3.3 Impact and friction sensitivity characterization

**Fig. 5:**
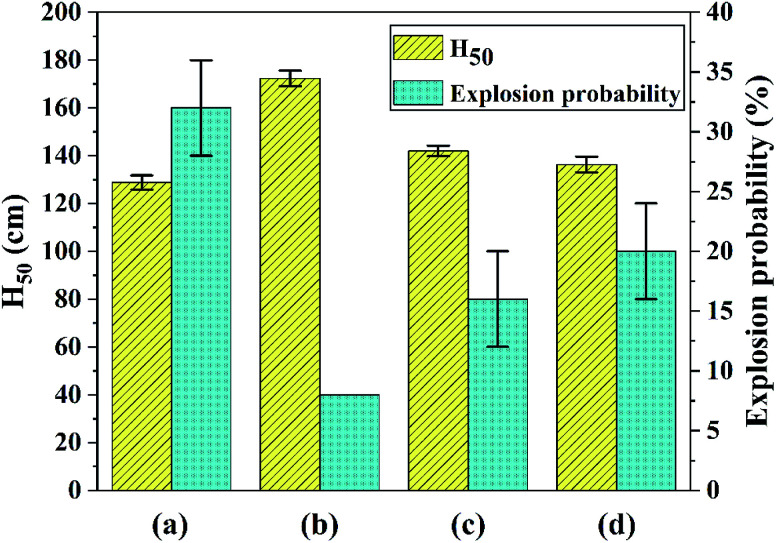
Results of impact and friction sensitivity test: (a) raw FOX-7, (b) FOX-7-1, (c) FOX-7-2, (d) FOX-7-3.

The samples (30 ± 1 mg) were subjected to an impact of a hammer (2 ± 0.002 kg) at various heights using an up-and-down method to investigate the safety performance of the explosive particles, and the critical drop height of 50% explosion probability (*H*_50_) was calculated (three tests per sample). The friction sensitivity of each product was tested under the same experimental conditions with 25 samples per group, and the explosion probability was calculated (three groups per sample). The number of explosions of each sample was recorded and the friction sensitivity is expressed by the arithmetic mean of the ratios of the number of explosions to the number of tests (which is 25) in each group. Results of impact and friction sensitivity test are shown in Fig. 5. At the same time, the data error bars in the sensitivity test are marked in Fig. 5.

As shown in Fig. 5, the *H*_50_ of raw FOX-7 (a) is (129 ± 3) cm, which is consistent with published work^1,23^ within a certain margin of error. And the *H*_50_ of products (b, c and d) were (172 ± 3) cm, (142 ± 2) cm and (136 ± 3) cm, respectively. An obvious increase of *H*_50_ for each product (43 cm, 13 cm and 7 cm, respectively) suggested that the ultrafine explosive particles were less sensitive than raw FOX-7. The hot-spot theory is used to explain the explosion of explosive particles under impact. When impacted, the gaps between explosive particles will undergo adiabatic compression to transform into many hot spots, which can lead to burning or explosion. As the particle size decreases, the specific surface area increases. When the explosive is subjected to an external impact load, the force is rapidly transmitted along the surface of the explosive particles, and the external force is dispersed to more surfaces, and the force exerted on the unit surface is reduced. And the impact sensitivity is greatly reduced when fewer hot spots are formed.

The explosion probability of raw FOX-7 was (32 ± 4)% and that of the products were reduced to 8%, (16 ± 4)% and (20 ± 4)% respectively, corresponding to Fig. 5. Explosive particles produced by mechanophysical milling (FOX-7-1 and FOX-7-2) had an ellipsoidal shape, leading to fewer hot spots during friction process and their friction sensitivity were lower than FOX-7-3 prepared by recrystallization, which is rod-shaped and its crystal structure is not dense and easy to break, resulting in a higher friction sensitivity. It can be concluded that an efficient desensitization effect had been achieved for the ultrafine FOX-7 particles.

### 3.4 Thermal analysis

**Fig. 6:**
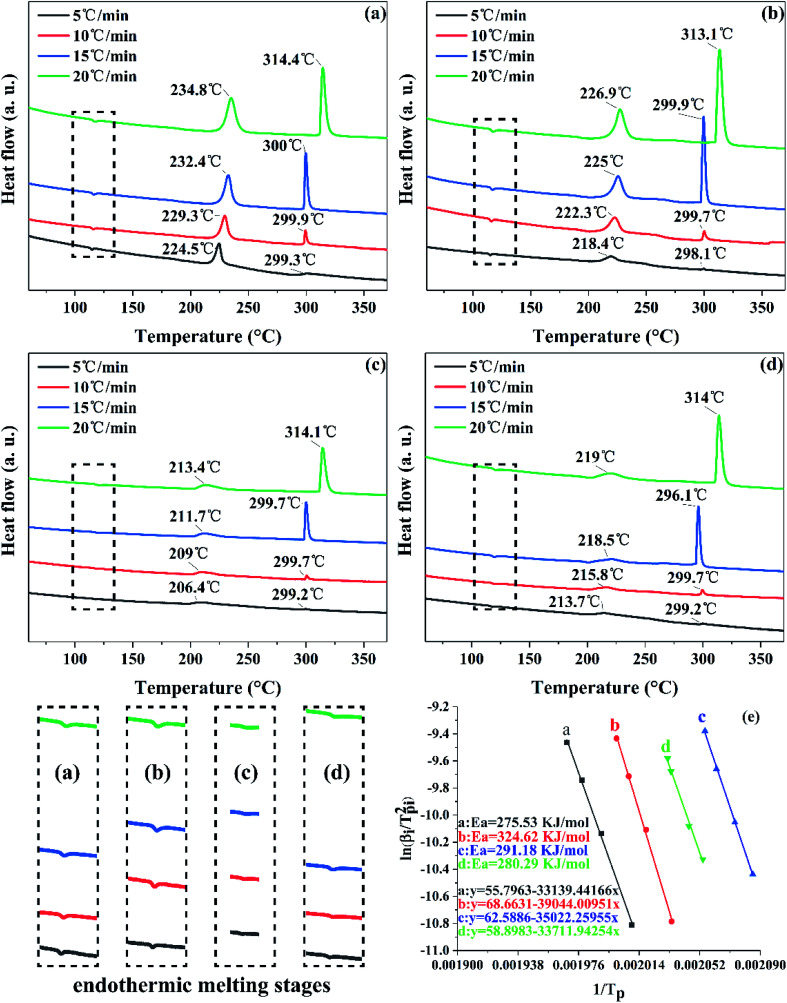
DSC measurement of the decomposition kinetics: (a) raw FOX-7, (b) FOX-7-1, (c) FOX-7-2, (d) FOX-7-3 and (e) Kissinger's plot of raw FOX-7 and products.

**Fig. 7:**
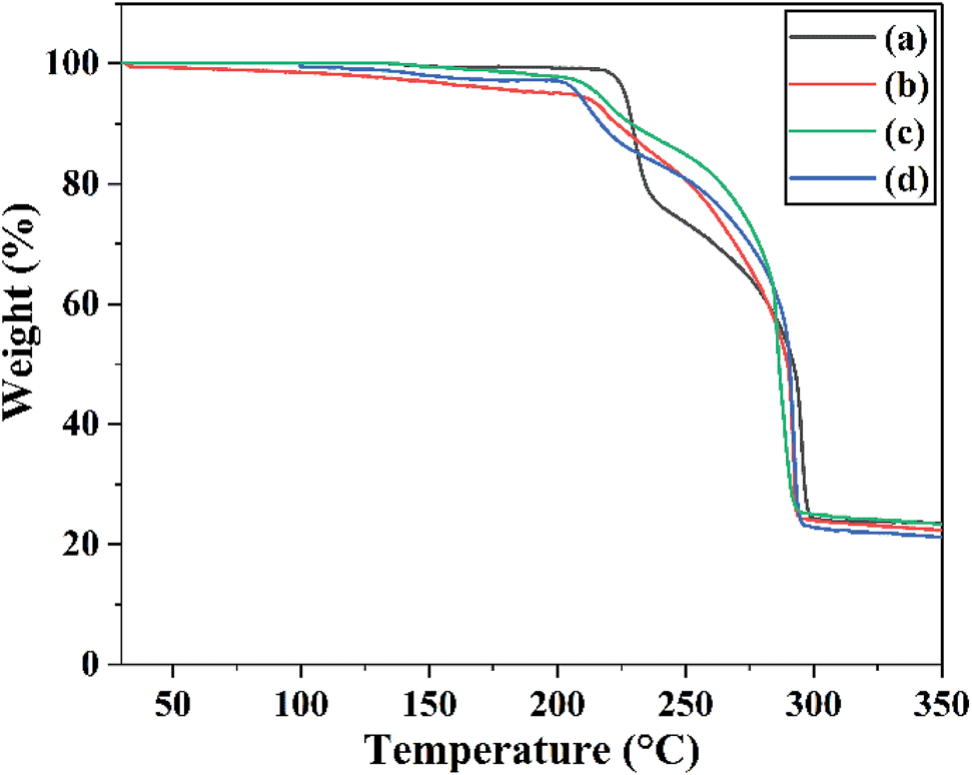
TG curves of (a) raw FOX-7, (b) FOX-7-1, (c) FOX-7-2 and (d) FOX-7-3 between 30 °C and 350 °C at a heating rate of 20 °C min^−1^.

The exothermal reaction is another important property. Differential scanning calorimetry (DSC) was used to investigate the thermal properties of raw FOX-7 and products. The DSC curves at different heating rates and TG curves of raw FOX-7 and products are shown in Fig. 6 and 7 respectively.

As we can see in Fig. 6, there are three distinct stages in the decomposition process of both raw FOX-7 and products: (1) endothermic melting stage (at 110–125 °C), (2) the first exothermic decomposition stage (at 210–230 °C) and (3) the second exothermic decomposition stage (at 290–310 °C). The first decomposition peak is caused by the rearrangement of nitro-to-nitrite in the FOX-7 molecule, bringing about the destruction of the conjugated system and hydrogen bonds,^24^ and the break of the carbon skeleton in the FOX-7 molecule leads to the second decomposition peak.^8^ It can also be seen from Fig. 6 that the decomposition peak temperature of the ultrafine FOX-7 was lowered compared with raw FOX-7, which can be attributed to an easier decomposition of ultrafine molecules at lower temperature because of more ability of atomic vibration, surface energy and the capacity of heat transmission.^25^

For each sample, the decomposition peak temperature and the heating rate were substantially positively correlated. And the second exothermic peak was higher than the first one when the heating rate was faster (15 °C min^−1^ and 20 °C min^−1^). Otherwise the first exothermic peak was higher than the second one (when the heating rate was slower such as 5 °C min^−1^ and 10 °C min^−1^). The advance of the highest exothermic peak may be due to a slower heating rate. Fig. 7 showed the TG curves of raw FOX-7 and products. Two obvious stages of weight loss resulted from exothermic decomposition can be observed and the position of the decomposition peak was consistent with the DSC curves. Moreover, there was no obvious inflexion point in the TG curves of ultrafine particles, and as-prepared ultrafine particles had higher decomposition rates compared to raw materials. These results are in good agreement with former studies.^6,8^

The Kissinger equation (eqn (1)) was enlisted to calculate the *E*_a_ (apparent activation energy) and *A* (pre-exponential factor) of raw FOX-7 and products, using the exothermic peak temperatures at different heating rates, respectively.1
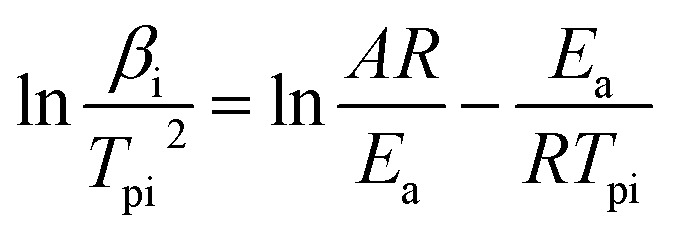
where *β*_i_ is the heating rate (in kelvin per minute); *T*_pi_ is the exothermic peak temperature in the DSC curve (in kelvin); *A* is the pre-exponential factor; *R* is the gas constant (8.314 J mol^−1^ K^−1^); *E*_a_ is the apparent activation energy (in J mol^−1^).

The activation energy (*E*_a_) and the pre-exponential factor (*A*) can be calculated by eqn (1), using the exothermic peak temperature (*T*_pi_) at different heating rates (*β*_i_), respectively. All the results are presented in Table 2.

**Table 2:** Thermal decomposition kinetic parameters of raw FOX-7 and products

Sample	*E*_a_/kJ mol^−1^	ln *A*	*R* ^2^
Raw FOX-7	275.53	59.30	0.99
FOX-7-1	324.62	72.33	0.99
FOX-7-2	291.18	66.14	0.99
FOX-7-3	280.29	62.42	0.99

Fig. 6(e) shows the Kissinger's plots of all samples and the results presented good linear relationships (*R*^2^ > 0.99) and the close plots may mean that the samples undergo similar decomposition reaction.^13^Table 2 shows that the activation energy of raw FOX-7 and products were 275.53 kJ mol^−1^, 324.62 kJ mol^−1^, 291.18 kJ mol^−1^ and 280.29 kJ mol^−1^, respectively. The physical meaning of apparent activation energy is the difficulty of the initiation of decomposition or deflagration. Thus, the higher apparent activation energy of ultrafine FOX-7 suggested that the decomposition reaction of ultrafine FOX-7 particles become difficult and had a better thermal stability. In this respect, the thermal stability of nano-FOX-7 particles (FOX-7-1) was most significantly improved.

## 4 Conclusion

In this paper, nano-FOX-7 particles were successfully prepared by mechanophysical milling and the safety of multi-scale FOX-7 particles were investigated. Particles with different sizes and morphologies were observed by FE-SEM. Impact and friction sensitivity of raw FOX-7 (20 μm) and ultrafine FOX-7 particles with different mean size (40 nm, 0.9 μm and 3.4 μm) were tested and thermal analysis of all samples was studied by DSC and TG. The results of sensitivity test showed that ultrafine particles were less sensitive and the apparent activation energy of ultrafine particles increased, suggesting their mechanical sensitivity and thermal stability were improved significantly.

## Conflicts of interest

There are no conflicts to declare.

## Supplementary Material

RA-009-C9RA03394G-s001

## References

[cit1] Latypov N. V., Bergman J., Langlet A., Wellmar U., Bemm U. (1998). Synthesis and reactions of 1,1-diamino-2,2-dinitroethylene. Tetrahedron.

[cit2] Bemm U., Ostmark H. (1998). 1,1-Diamino-2,2-dinitroethylene: a novel energetic material with infinite layers in two dimensions. Acta Crystallogr., Sect. C: Cryst. Struct. Commun..

[cit3] Ostmark H. , Langlet A., Bergman H., Wingborg N., Wellmar U. and Bemm U., FOX-7-a new explosive with low sensitivity and high performance, in 11th International Symposium on Detonation, Snowmass, CO, USA, 1998 -04-09, vol. 31

[cit4] Bellamy A. J. (2007). FOX-7 (1,1-Diamino-2,2-dinitroethene). Struct. Bonding.

[cit5] Zhonge C., Zhongyou L., Nan Y., Qing L., Du W. (2010). Safety Property of FOX-7 and HTPB propellants with FOX-7. Chin. J. Energ. Mater..

[cit6] Bing H., Zhiqiang Q., Fude N., Minhua C., Jing S., Hui H., Changwen H. (2010). Fabrication of FOX-7 quasi-three-dimensional grids of one-dimensional nanostructures *via* a spray freeze-drying technique and size-dependence of thermal properties. J. Hazard. Mater..

[cit7] Mandal A. K., Thanigaivelan U., Pandey R. K., Asthana S., Khomane R. B., Kulkarni B. D. (2012). Preparation of spherical particles of 1,1-diamino-2,2-dinitroethene (FOX-7) using a micellar nanoreactor. Org. Process Res. Dev..

[cit8] Bing G., Peng W., Bing H., Jun W., Zhiqiang Q., Guangcheng Y., Fude N. (2014). Preparation and characterization of nano-1,1-diamino-2,2-dinitroethene (FOX-7) explosive. New J. Chem..

[cit9] Yongxu Z., Dabin L., Chunxu L. (2005). Preparation and Characterization of Reticular Nano-HMX. Propellants, Explos., Pyrotech..

[cit10] Xiaolan S., Yi W., Chongwei A., Xiaode G., Fengsheng L. (2008). Dependence of particle morphology and size on the mechanical sensitivity and thermal stability of octahydro-1,3,5,7-tetranitro-1,3,5,7-tetrazocine. J. Hazard. Mater..

[cit11] Jie L., Wei J., Fengsheng L., Longxiang W., Jiangbao Z., Qing L., Yi W., Qing Y. (2014). Effect of drying conditions on the particle size, dispersion state, and mechanical sensitivities of nano HMX. Propellants, Explos., Pyrotech..

[cit12] Yi W., Jiang W., Xiaolan S., Guodong D., Fengsheng L. (2013). Insensitive HMX (octahydro-1,3,5,7-tetranitro-1,3,5,7-tetrazocine) nanocrystals fabricated by high-yield, low-cost mechanical milling. Cent. Eur. J. Energ. Mater..

[cit13] Baoyun Y., Chongwei A., Yuruo Z., Changkun S., Xiaoheng G., Jingyu W. (2018). One-Step Ball Milling Preparation of Nanoscale CL-20/Graphene Oxide for Significantly Reduced Particle Size and Sensitivity. Nanoscale Res. Lett..

[cit14] Xiaolan S., Yi W., Chongwei A. (2018). Thermochemical properties of nanometer CL-20 and PETN fabricated using a mechanical milling method. AIP Adv..

[cit15] Hao H., Jingyu W., Wenzheng X., Ruizheng X. (2009). Effect of Habit Modifiers on Morphology and Properties of Nano-HNS Explosive in Prefilming Twin-Fluid Nozzle-Assisted Precipitation. Propellants, Explos., Pyrotech..

[cit16] Jingyu W., Hao H., Wenzheng X., Yuruo Z., Bin L., Ruizheng X., Peiyong W., Ni Y. (2009). Prefilming twin-fluid nozzle assisted precipitation method for preparing nanocrystalline HNS and its characterization. J. Hazard. Mater..

[cit17] Baoyun Y., Chongwei A., Jingyu W., Xiaoheng G. (2017). Formation and properties of HMX-based microspheres *via* spray drying. RSC Adv..

[cit18] Chongwei A., Shuai X., Yuruo Z., Baoyun Y., Xiaoheng G., Jingyu W. (2018). Nano-HNS Particles: Mechanochemical Preparation and Properties Investigation. J. Nanomater..

[cit19] Chongwei A., Hequn L., Yuruo Z., Baoyun Y., Chuanhao X., Jingyu W. (2017). Preparation and Characterization of Ultrafine HMX/TATB Explosive Co-crystals. Cent. Eur. J. Energ. Mater..

[cit20] Hongwei Q., Rajen B. P., Reddy S. D., Victor S. (2015). Nanoscale 2CL-20·HMX high explosive cocrystal synthesized by bead milling. CrystEngComm.

[cit21] Xiaofeng S., Jingyu W., Xiaodong L., Chongwei A. (2014). Preparation and characterization of HMX/Estane nanocomposites. Cent. Eur. J. Energ. Mater..

[cit22] Huaqiang C., Li T., Bing H., Guangcheng Y., Debin G., Hui H. (2013). 1,1-Diamino-2,2-dintroethene (FOX-7) nanocrystals embedded in mesoporous carbon FDU-15. Microporous Mesoporous Mater..

[cit23] Anniyappan M., Talawar M. B., Gore G. M., Venugopalan S., Gandhe B. R. (2006). Synthesis, characterization and thermolysis of 1, 1-diamino-2, 2-dinitroethylene (FOX-7) and its salts. J. Hazard. Mater..

[cit24] Asta G., Lou M., Lulu H., Jerome K. (1999). Proposed Mechanism of 1,1-Diamino-Dinitroethylene Decomposition: A Density Functional Theory Study. J. Phys. Chem. A.

[cit25] Chaoyang Z., Qiang P., Liyan W., Xiaochuan W. (2010). Thermal Sensitivity of HMX Crystals and HMX-Based Explosives Treated under Various Conditions. Propellants, Explos., Pyrotech..

