# Supplementary material for: Preparation of multi-scale FOX-7 particles and investigation of sensitivity and thermal stability
Source: RSC Adv. 2019 Jul 4;9(36):21042–9. doi: 10.1039/c9ra03394g (PMC9065742; doi:10.1039/c9ra03394g)
Supplement: RA-009-C9RA03394G-s001 [file RA-009-C9RA03394G-s001.pdf]

Electronic Supplementary Material (ESI) for RSC Advances.
This journal is © The Royal Society of Chemistry 2019

Supplement Information for the Impact and Friction Sensitivity Test Setups

The experimental set-up for impact and friction sensitivity were shown in Figure 1 and 2 [1].

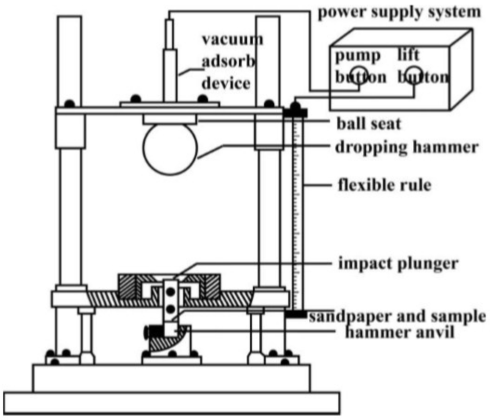

Figure 1 Experimental set-up for measuring impact sensitivity

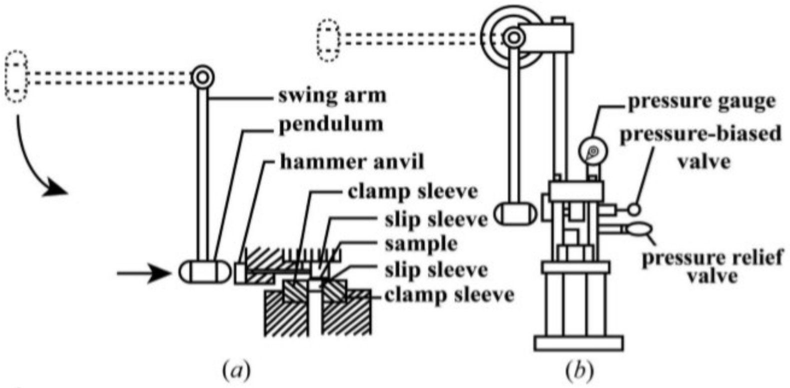

Figure 2 Experimental set-up for measuring friction sensitivity

The impact sensitivity test process: the weighed sample flowed out through a cone funnel at a constant speed and piled up on the center of the sandpaper to form a pyramid. The sandpaper with the sample was then carefully placed at the center of the anvil and the impact plunger was gently pressed against the sample. The power supply was turned on, the lift button pressed and the desired height of the ball seat adjusted (the height can be obtained from the flexible rule). The pump button was then turned on and the ball seat was pumped into a vacuum. The dropping hammer was adsorbed at the desired height, when it was near the ball seat. The pump button was turned off. The dropping hammer then became free and started to free fall until it hit the impact plunger. Then the sample on the sandpaper was impacted to explode by the impact plunger. The hammer anvil was used to buffer the residual impact after test. When the dropping hammer fell from the setting height, the sample exploded. This setting height is the special height H_50.

The friction sensitivity test process: the weighed sample was carefully placed between two slip sleeves and the clamp sleeve tightened. The pressure-biased valve was turned on and the pressured adjusted to 3.92 MPa. The hammer anvil was inserted and checked that it touched

the clamp sleeve. The pendulum was lifted and then released at a swaying angle of 90deg. Finally, the hammer anvil was taken out and the pressure relief valve was turned on. The friction sensitivity test was completed. Then the explosion probability of the sample was obtained by calculating the proportion of the total number of explosions to the total number of experiments.

[1] Yan L, Chongwei A, Jin L, and Jingyu W 2018 High-density HNIW/TNT cocrystal synthesized using a green chemical method *Acta Crystallographica Section B: Structural Science, Crystal Engineering and Materials* **74** 4
